# Effect of the Nature of Subsequent Environment on Oxytocin and Cortisol Secretion in Maltreated Children

**DOI:** 10.3389/fpsyt.2015.00173

**Published:** 2015-12-11

**Authors:** Sakae G. Mizushima, Takashi X. Fujisawa, Shinichiro Takiguchi, Hirokazu Kumazaki, Shiho Tanaka, Akemi Tomoda

**Affiliations:** ^1^Division of Developmental Higher Brain Functions, United Graduate School of Child Development, Osaka University, Kanazawa University, Hamamatsu University School of Medicine, Chiba University and University of Fukui, Fukui, Japan; ^2^Research Center for Child Mental Development, University of Fukui, Fukui, Japan

**Keywords:** child maltreatment, cortisol, oxytocin, residential care facility, hormones

## Abstract

Childhood maltreatment (CM), including abuse and neglect, is a crucial factor that distorts child development. CM is associated with alterations in numerous brain regions, and may be associated with hormonal dysregulation. This study aimed to investigate differences in secretion patterns of cortisol (CT) and oxytocin (OT) among children who experienced CM, children living in residential care facilities and in unstable environments. Among 38 maltreated children, 23 (mean age = 12.2 years, SD = 3.0) were categorized as “Settled” and 15 (mean age = 13.1 years, SD = 2.2) as “Unsettled.” Twenty-six age- and gender-matched (mean age = 12.6 years, SD = 2.1), typically developing (TD) children were also included. Clinical and psychological assessments, including IQ and trauma evaluations, were conducted for all participants. Age, gender, and full-scale IQ were used as covariates in hormone analysis. Two saliva samples were collected, one on awakening and the other at bedtime. There were significant differences in the awakening CT levels of the “Unsettled” group, and in bedtime OT levels in the “Settled” group as compared with TD children, and between CM groups. Furthermore, there was a significant difference in trauma-symptomatic depression scores between the “Settled” and “Unsettled” CM group. These results suggest that CT diurnal secretions tend to be reactive to current stress rather than previous experience. OT diurnal secretions are presumably hyper-regulated for coping with the environment to survive and thrive. By measuring salivary CT/OT diurnal patterns, hormonal dysregulation of CM children living in “Settled” environments and “Unsettled” environments was indicated.

## Introduction

Childhood and adolescence is a time of remarkable change in physical and psychological development, brain structure, and neuroendocrine function. Although the process is guided by genetic factors, the final form is determined by life experience and the living environment. From birth, parent–infant interaction modulates fundamental brain processes (1, 2), whereas more complex psychological development occurs in socializing with peers (3).

Adverse experience in the form of childhood maltreatment (CM) has been reported to bring several lifelong difficulties (4). For example, CM distorts attachment to, and sense of intimacy toward, “significant others.” Therefore, many maltreated children suffer from interpersonal communication problems throughout their lives. Thus, exposure to CM is acknowledged to impact mental health (5), causing repercussions in social environments, including parents, family, and local communities.

Childhood Maltreatment is also associated with alterations in the size (6, 7) and functional activity of a variety of brain regions (8), and with hormonal dysregulation (9). Prior studies (10–14) have demonstrated that CM leads to abnormal reactivity of hormones, such as cortisol (CT) and oxytocin (OT). These effects extend into adulthood (10–12) as well as influencing childhood (13, 14). Thus, early life stress is crucial in exacerbating mental health issues in the general population, as well as in psychiatric populations.

Cortisol is released in response to stress via the hypothalamic–pituitary–adrenal (HPA) axis. The secretion is regulated by capturing negative feedback relating to stress. CT levels are relatively high in the morning and decrease by evening (15, 16). Reportedly, children’s CT reactivity to stress is an important mediator of depression risk (17, 18). In addition, several studies of children with adverse childhood experiences (ACEs), such as institutionalized children in Romania (19, 20) and foster children (13, 21, 22), have indicated atypical “blunted” CT diurnal secretions. Prior CT studies related to CM have shown high CT levels in maltreated children with PTSD (23, 24) and elevated morning CT levels (25) in foster children who experienced severe emotional maltreatment. Although there exist inconsistencies regarding CT levels in maltreated children, previous studies of CM and depression in childhood suggest HPA axis dysregulation as a result of CT stress reactions.

The neuropeptide OT is secreted from the posterior pituitary gland, and has physiological functions during labor and lactation. There is a growing body of evidence that OT plays an important role in regulating social behavior in diverse species (26, 27). In humans, many studies have suggested that OT modulates mother–infant bonding, and OT receptors are involved in social behavior, including reproductive and maternal behaviors, as well as affiliation and attachment (28–31). It has been suggested that OT plays an important role in attachment formation or bonding with a “primary care-giver,” and individuals who have experienced that CM have atypical OT secretion patterns (32).

According to a Japanese government child welfare report in 2015 (33), more than 29000 children are institutionalized, such as in residential care homes, and 60% of those children have experienced CM. Before placing a child in institutionalized care, the child guidance center separately conducts several assessments of the children and their families; children are removed from their homes and placed with other children in temporary custody during the assessment. After the assessment, less than 30% of the children are placed in institutions [for more information about the institutionalized care system in Japan, see Suzuki and Tomoda (34)]. The remaining 67% of children return to their biological family homes.

Although the association between stressful events in childhood and abnormal hormonal levels are well established, few studies have examined the influence of CM on neuroendocrine function and pathways. Thus, developmental psychopathologists are highly interested in elucidating children’s HPA axis dysregulation, indexed via CT and/or OT reactivity, with two distinct literatures (13, 35) emerging on role of the environment following CM experiences, especially regarding differences in living environment.

This study explored the effect of the subsequent environment (i.e., stable or unstable placement) on salivary CT/OT secretion patterns in maltreated children. We hypothesized that differences in neuroendocrine function would be present when comparing those children in stable versus unstable environments. We divided the maltreated children into two groups according to their living environments, and analyzed salivary CT/OT secretion patterns as well as their association with psychological scales. Typically developing (TD) children acted as control subjects in our study.

## Materials and Methods

### Ethics Statement

The study protocol approved by the Ethics Committee of the University of Fukui (Assurance No. FU25-149). All participants’ parent(s) and the caregivers who took custody of the child gave written informed consent for participation in the study. The experimental protocol was conducted in accordance with the Declaration of Helsinki.

### Participants

This study involved 38 children who experienced CM, consisting of 19 boys and 19 girls. The children were subcategorized as “Unsettled” or “Settled.” The “Unsettled” group was defined as children who have been living with their biological parent(s), and also children living in residential childcare homes where the length of stay was less than 1 year. The reason for adopting the stay length of less than 1 year was based on our unpublished data from our previous study (34): we found that depressive mental state within the first year of institutionalization was significantly higher than that from the second year and more. Fifteen children (six boys and nine girls; mean age 13.1 years, SD 2.1) were categorized as “Unsettled.” The “Settled” group was defined as children who were settled in residential care homes instead of their original family homes. The duration within the settled environment ranged from a minimum of 1 year to a maximum of the child’s current age (the longest duration was 15 years). Twenty-three children (13 boys and 10 girls; mean age, 12.2 years, SD = 2.9) were categorized as “Settled.” Furthermore, 26 TD children (12 boys and 14 girls; mean age, 12.7 years, SD = 2.2) were included as healthy controls. The clinical patients who had experienced CM were recruited following their referral to the Department of Child and Adolescent Psychological Medicine at the University of Fukui Hospital via the local authority’s child guidance centers, other children were recruited to the CM group after their parents brought them to the hospital for medical attention related to difficulties in child rearing. Almost all CM group children were primarily screened regarding their ACE by the child guidance center before referral. The types of abuse experienced in the CM group were as follows: neglect (62%), physical abuse (35%), emotional abuse (16%), and sexual abuse (14%). Of the 38 children who experienced CM, 13 met the DSM-5 diagnostic criteria for reactive attachment disorder. In the public child welfare system in Japan, detailed duration of maltreatment experiences by a child is protected, along with the identity of the abuser, socioeconomic status, and home environment, so we could not access this information even though our purpose is academic, and the children’s identities would be protected.

The healthy age- and gender-matched TD controls were recruited from the general community in Fukui prefecture. They were recruited from the community through advertisements in a local newspaper and on our website homepage.

All participants’ race/ethnicity was Japanese. Participants were primarily screened by the MINI-International Neuropsychiatric Interview for children and adolescents (MINI-KID: Japanese version) (36, 37). None of the TD children had any history of Diagnostic and Statistical Manual of Mental Disorders, Fourth Edition, Text Revision (DSM-IV-TR) (38) Axis I Disorders, including developmental disorders, or any history of any form of abuse or drug abuse, head injury, any physical or abnormal developmental milestones, or fetal drug exposure. The intellectual ability of the participants was assessed by the Wechsler Intelligence Scale for Children-Fourth Edition (WISC-IV) (39).

### Assessment Measures

Previous studies have suggested that maltreated children have symptoms in common with neurodevelopmental disorders, such as attention-deficit hyperactivity disorder (ADHD) (40) and autism spectrum disorder (ASD) (41). In order to evaluate ADHD traits and ASD traits, the Japanese version of the ADHD Rating Scale-IV (42) and the Autism-Spectrum Quotient-children’s version (AQ) (43) were administered. The ADHD-RS (44) scale screens for children’s behavior during their routine activities. It consists of 18 questions, which are divided into two subcategories, consisting of nine inattention items and nine hyperactivity–impulsivity items. The AQ measures autistic traits of children (45) via 50 questions that are rated by the child’s parent or caretaker.

To assess behavioral problems, parents or caregivers completed the Strengths and Difficulties Questionnaire (SDQ) (46) which consists of 25 questions, split into 5 categories that assess children’s internalized (emotional and peer relationships) and externalized (conduct and hyperactivity) problems (“difficulties”). The prosocial score is excluded from the total score, because the former is categorized as a positive quality of the individual. SDQ data were obtained from the children and via their parent or caretaker as well. Depressive mental state was assessed via the Depression Self-Rating Scale for Children (DSRS-C: Japanese version) (47), which contains 18 questions that probe emotional and physical reactions associated with the current mood of children.

Traumatic experiences related to CM were evaluated by the UCLA PTSD Index for DSM-IV (UPID) (48), the Impact of Event Scale Revised (IES-R) (49, 50), and the trauma symptom checklist for children (TSCC) (51). UCLA PTSD Index consists of 47 questions, which follow the diagnostic criteria for PTSD from the DSM-IV (38). The evaluation usually takes 20–30 min and directly stimulates children; therefore, they are able to disclose their experiences naturally. IES-R is also structured by the diagnostic criteria of PTSD from the DSM-IV. This self-rating consists of 22 questions that ask how the individual’s thoughts and emotional state during the past week are influenced by previous trauma. TSCC consists of 54 questions, split into the six subcategories Anxiety, Depression, Anger, Post-traumatic Stress (PTS), Dissociation, and Sexual Concerns. These scales were applied during the structured interview by a licensed clinical psychologist (first author).

### Saliva Sampling

Awakening and bedtime saliva CT/OT data were obtained using Salivettes^®^ (Sarstedt, Rommelsdorft Corp., Germany). The awakening saliva sample was taken on waking, before breakfast (within 1 h of awakening). The bedtime saliva sample was taken before bedtime. The saliva sampling procedures were the same as those in our previous study (52). Before using Salivettes, instructions were given to all participants. Saliva samples were frozen and stored at −80°C in the laboratory. Salivary CT and OT levels were measured by enzyme-linked immune sorbent assay (ELISA). Before assay, each saliva sample was restored to room temperature. The assay was conducted using an all-in-one CT kit (Salimetrics™). These protocols are based on prior studies of children (53–55). The saliva samples were also used for OT analysis. After completing CT data analysis, the samples were lyophilized overnight and kept at −20º C to concentrate them two to four times. Afterwards, the dry samples were reconstructed in the assay buffer immediately before analysis, using an OT enzyme immunoassay commercial kit (Assay Designs Inc., Ann Arbor, MI, USA). The CT sample assay was duplicated, whereas the OT assay was executed in singlicate. The data were calculated using a SpectraMax^®^ (Molecular Device, Sunnyvale Corp., CA, USA) micro plate reader, according to relevant standard curves. Average intra- and inter-assay coefficients of variation were 9.6 and 8.6%, respectively.

### Statistical Procedure

Three different groups were defined, in order to determine characteristic features of maltreated children (“Unsettled,” “Settled,” and TD). Differences among the three groups in the clinical variables were assessed using one-way analysis of covariance (ANCOVA). Analysis of biological data (i.e., salivary CT and OT) involved repeated-measures two-way ANCOVA. Statistical analyses were conducted using IBM SPSS Statistics 21.0 software (SPSS Inc., Chicago, IL). All *p*-values were two-tailed; *p*-values less than.05 were considered significant.

## Results

### Demographic Data and Clinical Assessments

Table 1 shows the demographic and clinical characteristics of each group. The three groups were well matched in gender and age. ANOVA revealed that both CM groups (“Settled” and “Unsettled”) exhibited significantly lower full-scale IQ (FSIQ) scores than the TD group [*F* (2, 61) = 8.22, *p* < 0.0006]. For the clinical characteristics analysis among groups, FSIQ was adjusted using ANCOVA. Both CM groups (“Settled” and “Unsettled”) showed significantly higher scores than the TD group on ADHD-RS [*F* (2, 60) = 6.78, *p* = 0.0022], SDQ [Self-rating: *F* (2, 60) = 10.77, *p* < 0.0001, Parental-rating: *F* (2, 60) = 7.48, *p* = 0.0013]. However, for the DSRS-C, the “Unsettled” group scored significantly higher than the “Settled” group and TD children [*F* (2, 60) = 5.97, *p* = 0.0043]. There were no significant differences in AQ scores [*F* (2, 60) = 2.52, *p* = 0.0893]. These results suggest that CM group children (“Settled” and “Unsettled”) suffered from more behavioral and mood-related problems than the TD children, whereas the “Settled” CM group reported less severe difficulties than the “Unsettled” CM group.

**Table 1:** **Demographic data and clinical ratings of maltreated children (unsettled and settled) and typically developing children**.

Ratings	Unsettled	Settled	TD	Statistics	*p-*value
Number	15	23	26	–	–
Gender(M:F)	6:9	13:10	12:14	–	–
Age (mean ± SD)	13.1 ± 2.2	12.2 ± 3.0	12.1 ± 2.1	*F*_2,61_ = 0.60	ns
FSIQ	91.00 ± 13.3^b^	91.87 ± 9.6^b^	102.23 ± 9.0^a^	*F*_2,61_ = 8.26	<0.0006
ADHD-RS^d^	12.87 ± 10.6^a^	10.83 ± 9.8^a^	2.50 ± 2.4^b^	*F*_2,60_ = 6.78	<0.0022
AQ^a^	19.80 ± 5.7	20.22 ± 8.9	13.50 ± 5.7	*F*_2,60_ = 2.52	ns
SDQ (self-rating)^d^	17.00 ± 6.9^a^	15.70 ± 7.3^a^	7.50 ± 4.8^b^	*F*_2,60_ = 10.77	<0.0001
SDQ (parent-rating)^d^	14.00 ± 4.0^a^	13.70 ± 9.0^a^	6.04 ± 3.9^b^	*F*_2,60_ = 7.48	<0.0013
DSRS-C^d^	14.00 ± 7.3^a^	10.65 ± 6.7^b^	6.96 ± 4.7^b^	*F*_2,60_ = 5.97	<0.0043
IES-R^d^	25.47 ± 25.5^a^	16.30 ± 14.9^a^	2.65 ± 5.0^b^	*F*_2,60_ = 11.15	<0.0001
TSCC-anxiety^d^	54.07 ± 19.4^a^	48.13 ± 13.8^a^	39.42 ± 5.7^b^	*F*_2,60_ = 6.35	<0.0031
TSCC-depression^d^	52.87 ± 17.1^a^	43.04 ± 9.8^b^	37.19 ± 2.5^c^	*F*_2,60_ = 13.24	<0.0000
TSCC-anger^d^	48.80 ± 13.5^a^	43.65 ± 7.1^a^	36.81 ± 2.5^b^	*F*_2,60_ = 9.67	<0.0002
TSCC-post-traumatic stress^d^	50.60 ± 14.7^a^	44.74 ± 8.4^a^	37.58 ± 3.9^b^	*F*_2,60_ = 10.99	<0.0001
TSCC-dissociation^d^	48.47 ± 13.4^a^	47.26 ± 11.3^a^	37.50 ± 2.5^b^	*F*_2,60_ = 11.09	<0.0001
TSCC-sexual concerns^d^	41.47 ± 3.9	44.04 ± 9.7	38.85 ± 2.3	*F*_2,60_ = 1.80	ns

*FSIQ, full-scale IQ; ADHD-RS, attention-deficit hyperactivity disorder rating scale; AQ, autism-spectrum quotient-children’s version; SDQ, strength and difficulties questionnaire; DSRSC, depression self-rating scale for children (Birleson); IES-R, impact of event scale revised; TSCC, trauma symptom checklist for children. ^a^ > ^b^ (*p* < 0.05), ^a^ > ^c^ (*p* < 0.05), ^b^ > ^c^ (*p* < 0.05), ^d^Covariate items: FSIQ*.

For the trauma evaluation, according to the UPID (48), of the 38 subjects in the CM group, 3 met the full diagnostic criteria for PTSD, 6 were categorized as having partial PTSD, and 9 had a traumatic symptom above the UPID cut-off. Overall, 18 of 38 (47%) CM subjects had relatively mild to moderate PTSD symptoms. Both CM groups showed significantly higher scores than the TD group on the IES-R [*F* (2, 60) = 11.15 *p* < 0.0001] and most of TSCC sub-scales [Anxiety: *F* (2, 60) = 6.35, *p* = 0.0031; Anger: *F* (2, 60) = 9.67, *p* < 0.0002; PTS: *F* (2, 60) = 10.99, *p* < 0.0001; Dissociation: *F* (2, 60) = 11.09, *p* < 0.0001]. However, the “Unsettled” group generated significantly higher scores for TSCC-depression than the “Settled” group and TD group [*F* (82, 60) = 13.24, *p* < 0.00001]. This result suggests that “Unsettled” children tended to be more depressive than “Settled” CM and TD group children. No significant differences were found in the number of exposures to different types of maltreatment in the CM children [neglect; *F* (1, 36) = 2.97, *p* = 0.093, physical abuse *F* (1, 36) = 0.52, *p* = 0.477, psychological abuse *F* (1, 36) = 0.32. *p* = 0.578, sexual abuse *F* (1, 36) = 1.53, *p* = 0.224].

### Salivary Cortisol and Oxytocin

**Figure 1:**
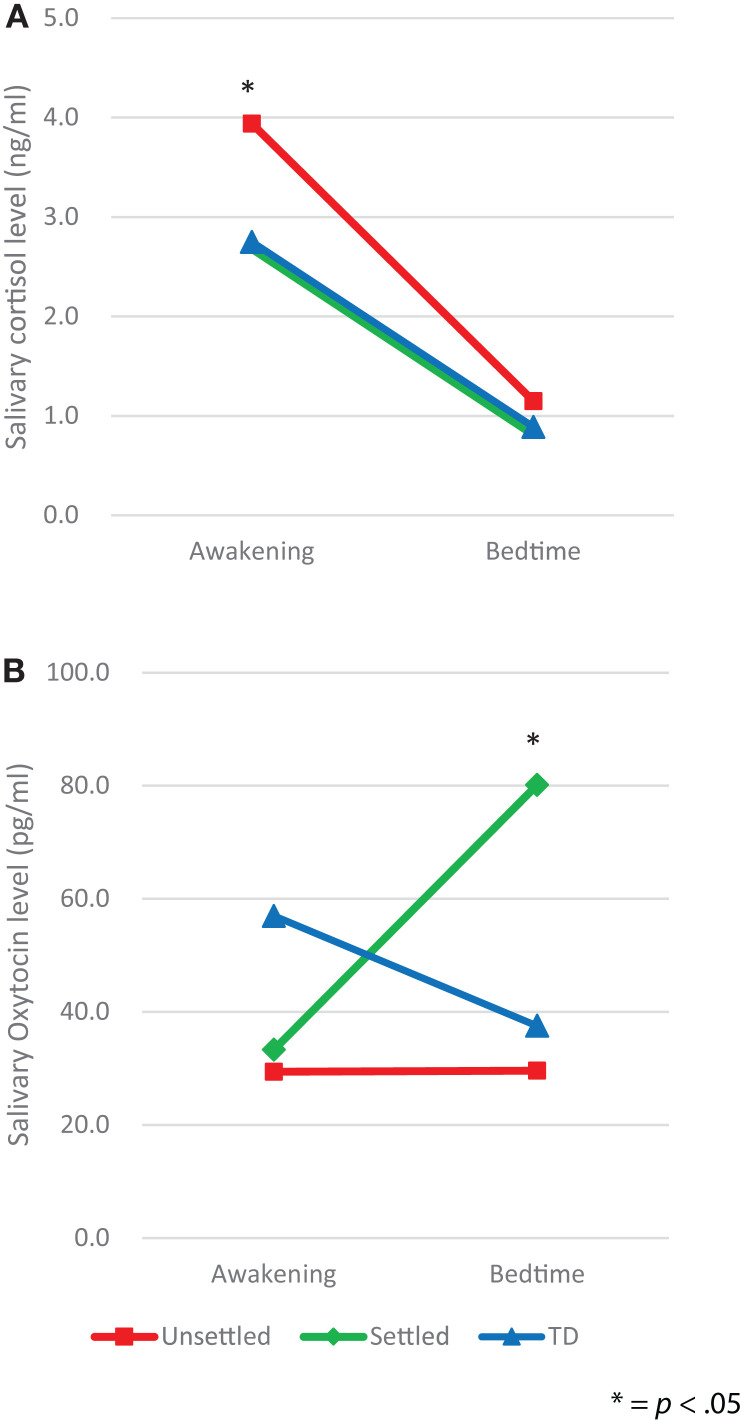
**Diurnal secretion of salivary hormones in maltreated children (unsettled and settled) and typically developing children: (A) Cortisol and (B) Oxytocin**.

We, first, explored differences between the CM and TD groups in salivary levels of CT and OT, by using ANCOVA with covariates consisting of age, gender, and FSIQ, which revealed no significant differences (*awakening CT, p* = *0.079, bedtime CT, p* = *0.333; awakening OT, p* = *0.119, bedtime OT, p* = *0.213*). Second, in order to examine differences in salivary levels of CT and OT between “Unsettled,” “Settled,” and TD groups, each salivary level was compared using repeated-measures two-way ANCOVA with covariates consisting of age, gender, and FSIQ; secretion slope (awakening and bedtime) as a within-participants factor, and environmental category (“Unsettled,” “Settled,” and TD) as a between-participants factor. For CT levels (Figure 1A), we found a main effect of the time of day [*F* (2, 58) = 5.18, *p* = 0.008]. This indicates that the average CT level was significantly higher on awakening than at bedtime. The main effect of environmental category (i.e., CM influence) was not significant, however, the interaction effect with time of day was significant [*F* (2, 58) = 5.18, *p* = 0.008], indicating that the “Unsettled” environment effect was greater on awakening than at bedtime. For OT levels (Figure 1B), there was a significant difference in the bedtime OT level between CM groups [“Settled” and “Unsettled”; *F* (2, 58) = 4.00, *p* = 0.05]. This result indicates that “Settled” children’s OT secretions markedly increased from awakening to bedtime.

Additional analyses were conducted to determine whether there were between-group differences in diurnal patterns of CT and OT secretion. For CT and OT secretions, the area under the curve (AUC) and diurnal slope (change in levels from awakening to the bedtime) were calculated, and group-wise effects investigated using between-participants one-way ANOVAs. For the AUC, there was no significant difference in CT and OT hormone levels among the groups. However, a significant difference in the CT slopes was found [*F* (2, 58) = 4.16, *p* = 0.02], as shown in Table 2, whereas the OT slopes did not differ among groups [*F* (2, 58) = 1, *p* = 0.374]. *Post hoc* analysis using Bonferroni correction indicated that the decline in CT level from awakening to bedtime was significantly larger in the “Unsettled” group than the TD children [*F* (2, 58) = 4.16, *p* = 0.019]. This result indicates that the “Unsettled” children’s CT secretion levels dropped sharply compared to the other groups (Figure 1A). There were no significant correlations between TSCC-depression scores and salivary CT or OT levels, adjusting FSIQ as a covariate (*N* = 64; CT awakening: *r* = 0.087, *p* = 0.501; CT bedtime: *r* = 0.084, *p* = 0.518; OT awakening: *r* = −0.101, *p* = 0.433; OT bedtime: *r* = −0.039, *p* = 0.764).

**Table 2:** **Cortisol and Oxytocin measures in maltreated children (unsettled and settled) and typically developing children**.

Measures	Unsettled	Settled	TD	Statistics	*p-*value
Cortisol AUC (ng/ml)^d^	29.96 ± 15.0	25.41 ± 8.3	26.08 ± 9.8	*F*_2,58_ = 0.58	ns
Oxytocin AUC (pg/ml)^d^	385.89 ± 274.9	784.71 ± 723.3	835.09 ± 886.4	*F*_2,58_ = 2.55	ns
Cortisol slope^d^	0.22 ± 0.2^a^	0.15 ± 0.1^b^	0.12 ± 0.1^b^	*F*_2,58_ = 4.16	<0.02
Oxytocin slope^d^	0.11 ± 1.2	−2.13 ± 5.9	0.33 ± 7.3	*F*_2,58_ = 1.00	ns

*Cortisol AUC, cortisol’s area under the curve; Oxytocin AUC, oxytocin’s area under the curve. ^a^ > ^b^ (*p* < 0.05)*.

*^d^Covariate items: age, gender, and FSIQ*.

## Discussion

The present study demonstrated distinct patterns of salivary levels of CT and OT secretions among maltreated children living in different environments (“Unsettled” and “Settled”), and TD children. There were significantly higher levels of awakening CT in the “Unsettled” group compared with the “Settled” group and TD group. Similarly, the “Unsettled” group had significantly higher DSRS-C scores than TD group, and higher TSCC-depression scores than the “Settled” and TD groups. These results suggest that children living in “Unsettled” environments may have hyper-regulation of the HPA axis as an adaptation to cope with their environment and this hyper-regulation may induce a depressive mental state. Moreover, our results also demonstrate that children in “Settled” environments have a marked increase in the bedtime salivary OT level compared with TD children. This result suggests that the formation of stable interpersonal relationships with facility staff and their peers may lead to atypical patterns of OT diurnal secretions. Thus, maltreated children living in different environments may differ in their hormonal dysregulation.

### Cortisol Studies

Our results are consistent with previous findings that higher cortisol is associated with depressive problems (56, 57). Bernard (13) conducted a similar comparative study of CT patterns in maltreated children who live with their birth parents and those in foster care. However, the results were in complete opposition to ours. This may be because Bernard’s study targeted very young children. According to Jessop’s (58) review, HPA axis development is not mature in children who are younger than 4 years of age. Prior studies have suggested that maltreated children’s CT is unusually low in the morning and slightly high in the evening (13, 19–22). However, children with PTSD (24, 59) tend to show an elevated or hyperactive pattern. Thus, a consistent picture of maltreated children’s CT secretion pattern is currently unavailable. Empirical studies (18, 25) have suggested hyperactivity in children who internalize problems. Our “Unsettled” children surely struggle with emotional burdens, unpredictable threats, and a lack of social support. They not only confront the concomitants of CM but also experience numerous stresses within their environments. By contrast, the “Settled” children stay in residential care facilities, protecting them from their prior CM experiences. Our “Settled” children’s CT secretion patterns were clearly quite similar pattern to those of TD children. This suggests that the CT reactivity prioritizes current stress exposure and depressive states, rather than previous early life stresses.

### Oxytocin Studies

A recent study showed an association between exposure to less severe forms of CM and higher OT concentrations, suggesting that adversity may enhance OT secretions, in order to cope with the social environment (60). Although our result was partially consistent with their finding, we only found higher levels of OT at bedtime in the “Settled” CM group. One possible explanation for the higher levels of OT may be related to alteration of the social environment of residential care facilities following the adverse experience.

Previous studies have suggested that OT levels are elevated by social interaction with “Significant others,” such as parents (61, 62). In addition, OT levels after social exposure among maltreated children were reported to be higher than those in healthy controls (35). A meta-analysis found that oxytocin administration showed greater attenuation of the cortisol response in laboratory tasks that strongly activated the HPA axis, suggesting that oxytocin may play an important role in HPA dysfunction associated with psychopathology (63). After removal from their homes, “Settled” group children were living in childcare facilities with other children, and without ongoing child maltreatment exposure. These “Settled” children may have adapted to the stable environment in order to survive and thrive, explaining the hyperactivity of OT secretions.

### Biological Clocks

Hormone secretion is regulated by circadian rhythms, which are driven by endogenous biological clocks. A master circadian clock is present in the suprachiasmatic nucleus of the anterior hypothalamus (64, 65), which regulates many neuroendocrinological, physiological, and behavioral processes. The alteration in circadian time structure likely plays an important role in the pathogenesis of hormone levels. The present study highlights the need to consider the plight of maltreated children, which is associated with alterations to the HPA axis that affect maltreated children differently in terms of the living environment. CM likely diminishes a person’s ability to cope with stress, possibly placing them at risk for psychopathology and ill health. The effect of CM on the circadian rhythm can alter neuroendocrine regulation, produce or diminish HPA axis activity, and change the strength of post-traumatic growth (12, 66, 67). All these findings suggest that early adversity affects hormonal function, even after several years have elapsed to relieve the stress exposure and to adapt to the social environment.

### Study Strengths and Limitations

A strength of our study is the comprehensive assessment of our participants, by obtaining FSIQ and psychological scale data, and a full trauma evaluation in order to understand CM’s developmental influence on CT and OT. The present study indicated lower IQ in maltreated children than in TD children, justifying our use of FSIQ as a covariate in the psychological scale and salivary sample analysis. To our knowledge, this is the first study of basal salivary CT and OT levels comparing CM children in “Unsettled” and “Settled” environments.

Our study has several limitations, the first of which is our modest sample size. Therefore, the relationship between the subsequent environments of maltreated children and their hormonal secretion remains tentative. In the preliminary results of this study, a positive dose–response association between the number of CM types and both hormone levels was not observed. Therefore, the relationship between their levels and abuse history remains unclear. Replication of our result with a larger sample would be worthwhile. Second, we did not control for pubertal influences in the participants. Pubertal transitions (68) are potentially important, because hormonal development is dramatically changed during this period. Previous studies have suggested that child sexual abuse, physical abuse, and a stressful family environment may accelerate sexual maturation (69, 70). Third, this was not a longitudinal study and, therefore, resilience was not assessed in our study groups; no information related to outcomes in late adolescence is available for maltreated children. However, the resilience and post-traumatic growth issues of those children must be very important from a physiological, as well as a psychopathological, point of view (71, 72). Fourth, the subjects’ socioeconomic status (SES) was not examined in this study because of inadequate information on parents’ data among children in residential care facilities. While childhood SES has been associated with neurocognitive and brain development, recent evidence from neurodevelopmental cohort studies has suggested that SES might play an important role in adolescent neuroendocrine development (73, 74). Finally, we primarily screened severity of CM using ACE (75), but we did not find any significant differences of environmental factors between CM groups. If we were able to obtain onset and duration of abuse information for those children more precisely, this would better indicate cause and effect relationships within child maltreatment. These limitations should be addressed in the future studies.

## Conclusion

Our study revealed dysfunction in the hormonal secretions of CM children living in “Settled” environments and “Unsettled” environments as compared with TD children. Specifically, we found significantly higher awakening CT secretions in children in unstable environments, and significantly higher bedtime OT secretions in children in the residential care facilities. Regarding psychological evaluations, trauma-related depression scores in the “Unsettled” CM group were significantly higher than those in “Settled” CM and TD children. Our study indicates how early adversity influences children’s neuroendocrine regulation, physically and psychologically. Longitudinal studies with larger samples are needed to investigate whether an intervention program or treatment is associated with stable changes in hormonal secretions related to stable mental health.

This study did not directly investigate the neural mechanisms underlying any effects. However, hormone release is based on neural function. Combining our experimental paradigm with neuroimaging techniques is necessary to elucidate the association between neural function and hormone release. Such paradigms would provide valuable clarification of cause–effect relationships between development and environment.

## Conflict of Interest Statement

The authors declare that the research was conducted in the absence of any commercial or financial relationships that could be construed as a potential conflict of interest.
