# Corrigendum: Effect of the nature of subsequent environment on oxytocin and cortisol secretion in maltreated children

**DOI:** 10.3389/fpsyt.2024.1451873

**Published:** 2024-08-05

**Authors:** Sakae G. Mizushima, Takashi X. Fujisawa, Shinichiro Takiguchi, Hirokazu Kumazaki, Shiho Tanaka, Akemi Tomoda

**Affiliations:** ^1^ Division of Developmental Higher Brain Functions, United Graduate School of Child Development, Osaka University, Kanazawa University, Hamamatsu University School of Medicine, Chiba University and University of Fukui, Fukui, Japan; ^2^ Research Center for Child Mental Development, University of Fukui, Fukui, Japan

**Keywords:** child maltreatment, cortisol, oxytocin, residential care facility, hormones

In the published article, there was an error in **Materials and Methods**, *Participants*, Paragraph 1. The number of participants diagnosed with reactive attachment disorder was mistakenly omitted. This sentence previously stated:

“The types of abuse experienced in the CM group were as follows: neglect (62%), physical abuse (35%), emotional abuse (16%), and sexual abuse (14%).”

The corrected sentence appears below:

“The types of abuse experienced in the CM group were as follows: neglect (62%), physical abuse (35%), emotional abuse (16%), and sexual abuse (14%). Of the 38 children who experienced CM, 13 met the DSM-5 diagnostic criteria for reactive attachment disorder.”

The authors apologize for this error and state that this does not change the scientific conclusions of the article in any way.

